# Nitrogen Isotopes Suggest Sex‐Based Diet Differences on the Breeding Grounds for a Sexually Monomorphic Migratory Passerine

**DOI:** 10.1002/ece3.71720

**Published:** 2025-07-16

**Authors:** Autumn R. Iverson, Renée L. Cormier, Diana L. Humple, Thomas P. Hahn, Jessica Schaefer, Elisha M. Hull, Walter H. Sakai, Samuelle Simard‐Provençal

**Affiliations:** ^1^ Department of Animal Science University of California Davis California USA; ^2^ Point Blue Conservation Science California USA; ^3^ Department of Neurobiology, Physiology and Behavior University of California Davis California USA; ^4^ Thousand Oaks California USA; ^5^ Vancouver Island University Nanaimo British Columbia Canada

**Keywords:** diet, foraging, migratory species, nitrogen, passerine, stable isotope

## Abstract

Differential foraging by sex can have important implications for understanding the ecology of a species. This can be especially difficult to study through observations alone in sexually monomorphic species, such as the Golden‐crowned Sparrow ( *Zonotrichia atricapilla* ), and for species in remote areas. We used nitrogen and carbon stable isotope analysis to determine the relative trophic position between the sexes for 73 individual Golden‐crowned Sparrows, a migrant songbird species with little known diet information from remote breeding locations of Alaska and northwestern Canada. We found no evidence of differences in feather δ^13^C between the sexes suggesting similar habitat use, but we found an average 0.3‰ increase each year that may indicate increasingly water stressed habitats. We found that females had significantly higher values of feather δ^15^N (mean 5.4‰; mean for males 4.5‰) after accounting for year and feather collection location and in a subset of GPS‐tagged birds with known breeding locations, after accounting for year, breeding latitude, elevation, and distance to shoreline. We infer that females may be foraging on more food items from a higher trophic level than males on breeding grounds, which may reflect a physiological need to replace lost nutrients from nesting. If females rely on insects during the breeding season, then their success will be tied to insect populations which are generally experiencing large declines. Additionally, we provide mass and wing chord measurements from genetically sexed individuals to add to currently low published sample sizes for this monomorphic species.

## Graphical abstract

Differential foraging by sex can have important implications for understanding the ecology of a species. Using stable isotope values from feathers, we found evidence that female Golden‐crowned Sparrows are eating at a higher trophic level than males during the breeding season molt, which may reflect a physiological need to replace lost nutrients from nesting.

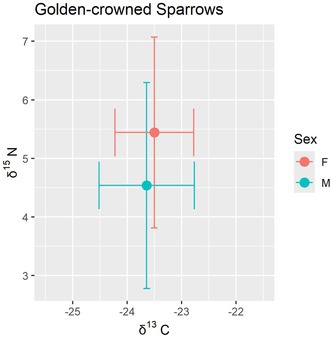

## 1 Introduction

Inter‐ and intraspecific variation in diet can have important implications for populations, contributing to competition among species or populations and/or differences in exposure to anthropogenic threats. In birds, differences in foraging strategy can help explain spatial overlap of similar species (Hipfner et al. 2013), and within a species, sex‐based differences in foraging can be linked to differences in physiology, body condition, and/or body size that can have implications for movement ecology and conservation (e.g., seabirds: Phillips et al. 2017; Merlin *Falco columbarius* : Bourbour et al. 2021; shorebirds: Nebel and Thompson 2011). While many studies on sex‐based foraging differences focus on seabirds, shorebirds, or raptors, there can be important ramifications for passerines as well. For example, sex‐based habitat segregation in wintering American Redstarts ( *Setophaga ruticilla* ) and Black‐and‐white Warblers ( *Mniotilta varia* ) results in females with lower body condition and survival, leading to negative carry‐over effects (Cooper et al. 2021; Marra and Holmes 2001).

Both carbon (δ^13^C) and nitrogen (δ^15^N) stable isotopes can provide insights into foraging ecology and have been used since the 1970s for this purpose (reviewed in Kelly 2000). For example, carbon stable isotope analysis can indicate broad differences in habitat type used between individuals, such as between areas dominated by C_3_ or C_4_ photosynthesis and/or associated with pelagic or benthic marine food webs (Forero et al. 2005; Hobson et al. 1994; Kelly 2000) and nitrogen stable isotope analysis can elucidate trophic levels, with the δ^15^N values of a consumer generally enriched by 3‰–4‰ (parts per thousand) compared to its food source (Post 2002). Even without the isotopic signatures of the food source and therefore no ability to determine the absolute trophic position (Post 2002), comparing relative values of carbon and nitrogen stable isotopes can still be informative when comparing across different groups to assess potential niche similarity (e.g., Lorenz et al. 2020).

The Golden‐crowned Sparrow ( *Zonotrichia atricapilla* ) is a sexually monomorphic migratory passerine that breeds in remote areas of Alaska and northwestern Canada, where very little is known about its diet (Pandolfino et al. 2025). As part of a larger study on the migration and breeding ecology of this species using GPS tracking (Iverson et al. 2023, 2024), we collected feather samples to investigate the foraging ecology of Golden‐crowned Sparrows using δ^13^C and δ^15^N stable isotope analysis. While we did not collect samples of diet items from the breeding range, which would enable the determination of a specific trophic position, we sought to understand the relative difference between the sexes using δ^13^C and δ^15^N values from feathers collected on their wintering range but grown on their breeding grounds.

## 2 Material and Methods

### 2.1 Feather Sampling

**FIGURE 1:**
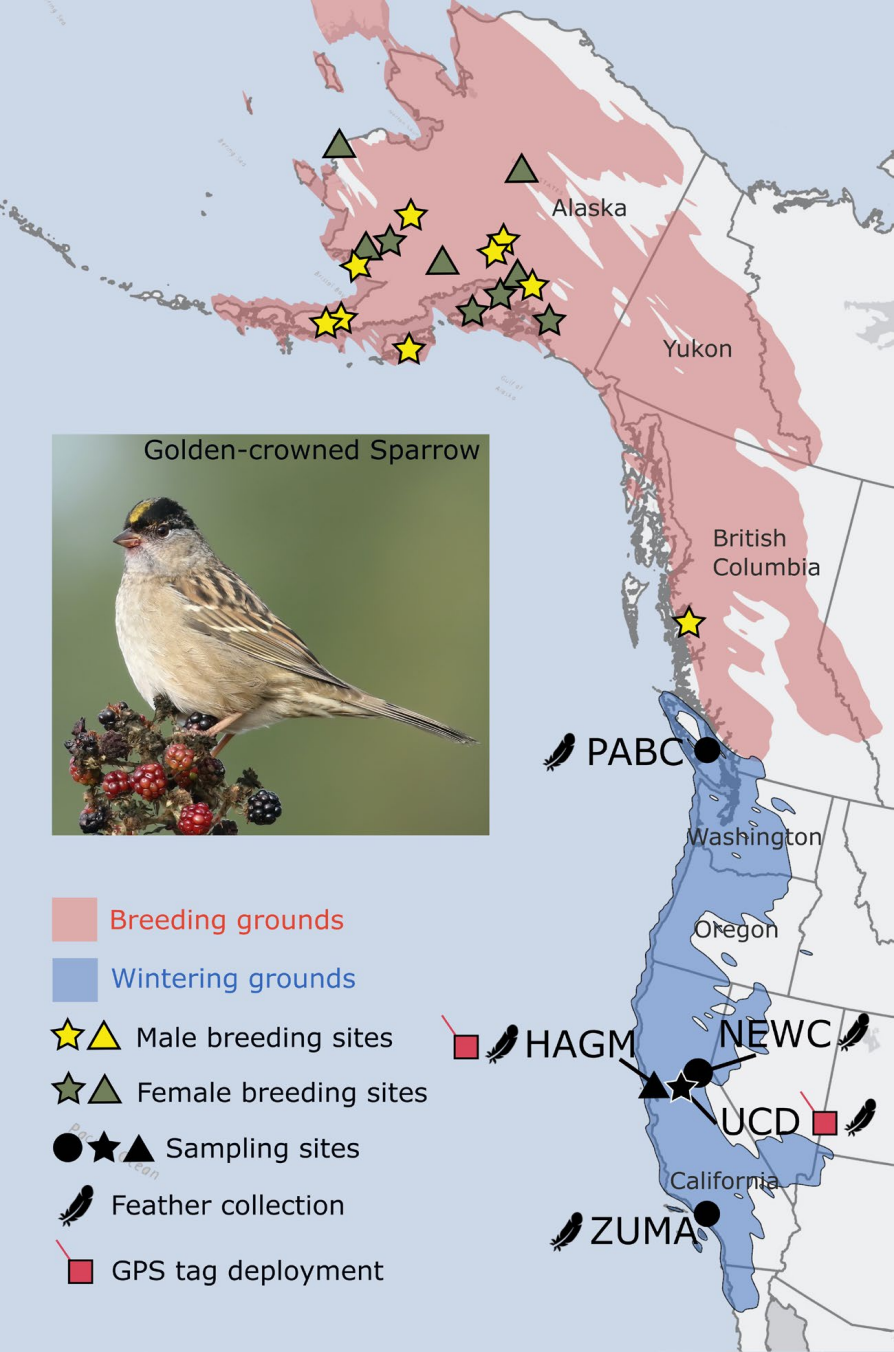
Study sites (black symbols) for Golden‐crowned Sparrows ( *Zonotrichia atricapilla* ; inset) where feathers were collected on the wintering grounds (blue color) during winters 2019–2021 (breeding range shown in salmon color) and associated breeding locations for tagged birds (from Iverson et al. 2024; shape designates where the bird was tagged and color designates the sex). PABC = Parksville, British Columbia (Canada); UCD = University of California Davis (USA); HAGM = Hagmaier Ranch, Olema Valley, Point Reyes National Seashore (USA); ZUMA = Zuma Canyon, Malibu (USA); NEWC = Newcastle, California; see Methods for specific coordinates. Feather icons denote where feathers were collected and tag icons denote where GPS tags were deployed. Ranges based on estimated ranges from eBird (Fink et al. 2023). Political boundaries from the Commission for Environmental Cooperation (www.cec.org). Inset photo credit: Liam Singh.

We used Potter traps baited with seed (millet or mixed seeds) and/or mist nets to capture birds. Birds were caught at five study sites (Figure 1) including (1) from 16 to 24 Feb 2021 in Parksville on Vancouver Island, British Columbia, Canada (PABC, 49.33, −124.28), and at 4 California, USA sites: (2) from 19 Feb 2019 to 30 Jan 2021 at the University of California Davis campus, Yolo County (UCD, 38.53, −121.78), (3) from 3 Jan 2019 to 9 Jan 2021 at Hagmaier Ranch in Olema Valley, Point Reyes National Seashore, Marin County (HAGM, 37.971, −122.731), (4) from 1 Jan to 7 Mar 2020 at Zuma Canyon in Malibu, Los Angeles County within the Santa Monica Mountains National Recreation Area (ZUMA, 34.0, −118.4), and (5) from 7 Mar to 8 Mar 2020 at a landscaped yard adjacent to an oak woodland with a nearby riparian area in Newcastle, California (NEWC), in Placer County (38.88, −121.19) in the foothills of the Sierra Nevada mountains.

In songbirds, molt of primary feathers generally occurs immediately after breeding at a nearby molt location and happens over a 1–2 month period, though long‐distance migrants and birds breeding in northern latitudes may molt over shorter periods (Howell 2010; Jenni and Winkler 1994). Rectrices (tail feathers) are generally replaced during molting of primary and secondary feathers (Pyle 2022). Golden‐crowned Sparrows have a complete prebasic molt (i.e., all feathers are replaced) that occurs on the breeding grounds (primarily July–September; Pandolfino et al. 2025) and a prealternate molt (i.e., some feathers are replaced) on wintering grounds that may include 1–2 central rectrices (Pyle 2022). While there is always the possibility of adventitious loss of a tail feather due to predator encounters or fights, and there may be occasional suspension of the prebasic molt until reaching wintering grounds (see Pandolfino et al. 2025), we collected the outermost rectrices (1 on each side) to increase our chances of collecting feathers grown during the post‐breeding prebasic molt and therefore that reflect growth at the breeding grounds. Songbird feathers can grow 2–5 mm/day (Howell 2010), and the amount sampled for stable isotope analysis was a 1 cm section 3–4 mm from the tip (see Stable isotope analysis section below), therefore representing ~2–5 days of growth. Once feathers are formed, isotopic turnover does not occur (Hobson and Clark 1992).

We collected feathers in the months of October–March from 162 individuals at five study sites across the Golden‐crowned Sparrow wintering range (western regions of Canada and the United States) from 2019 to 2021 and deployed 50 GPS tags as part of a larger migration study on this species (Iverson et al. 2023, Iverson et al. 2024; Figure 1). The previous study on breeding home ranges for GPS‐tracked Golden‐crowned Sparrows showed that individuals stayed on average within 0.4 km^2^ during the entire breeding season before leaving for fall migration and that males and females departed the breeding grounds around the same time, on average in early September (Iverson et al. 2024). We collected 8–10 contour feathers for genetic sexing and two outer tail feathers (rectrices) for stable isotope analysis. Feather sampling for GPS‐tagged birds happened upon tag retrieval for all sites and for other birds upon initial capture during the study window. In addition to feather sampling, we banded each individual with a federal band (and sometimes color bands) and recorded various measurements including body mass and wing chord length.

### 2.2 Sexing Birds

Golden‐crowned Sparrows cannot be reliably sexed by plumage (Pyle 2022), so we used the contour feather samples for genetic sexing. We sent contour feather samples to Animal Genetics Inc. (Tallahassee, FL), and they determined the sex of 60 individuals, including all birds with retrieved GPS tags (*n* = 23). Three birds were tracked with GPS twice (see related study Iverson et al. 2024) and the only data (feathers, measurements, breeding locations) associated with their first capture are included in this paper.

Due to funding limitations, we were only able to genetically determine sex for a subset of the birds with stable isotope information, leaving 102 birds with δ^15^N values but unknown sex. Published wing chord lengths differ slightly between the sexes, with female wing chord length ranging from 71 to 81 mm (*n* = 100) and male wing length ranging from 76 to 84 mm (*n* = 100; Pyle 1997; lower range is the same but upper range is reported as 1 mm less for each sex in Pyle 2022 also with *n* = 100). As published wing chord measurements for males and females overlap and are only for 100 birds, we (1) assessed whether published measurements reflected the values in our population, and (2) tested whether wing chord lengths differed between the males and females in our sample of birds with genetically determined sexes by running an ANOVA using the *stats* package in R (R Core Team 2024) with wing chord length as the response and sex as the predictor. As we found that published wing chord ranges encompassed the wing chord values we found in our population for females and the lower range for males (see Results), we used the longest published wing chord length for females (81 mm) and the shortest for males (76 mm) to sex birds without genetic testing. Also, to account for potential variation in wing chord measurements taken by banders, we added an additional 2 mm buffer, and therefore classified any birds with wing chord lengths ≤ 74 mm as female (2 mm less than smallest measurement for males), and any birds with wing chord lengths ≥ 83 mm as male (2 mm more than the largest measurement for females). We also conducted an ANOVA to test for differences in mass between genetically sexed individuals because morphometric data on sex differences for this species is limited.

### 2.3 Stable Isotope Analysis

**FIGURE 2:**
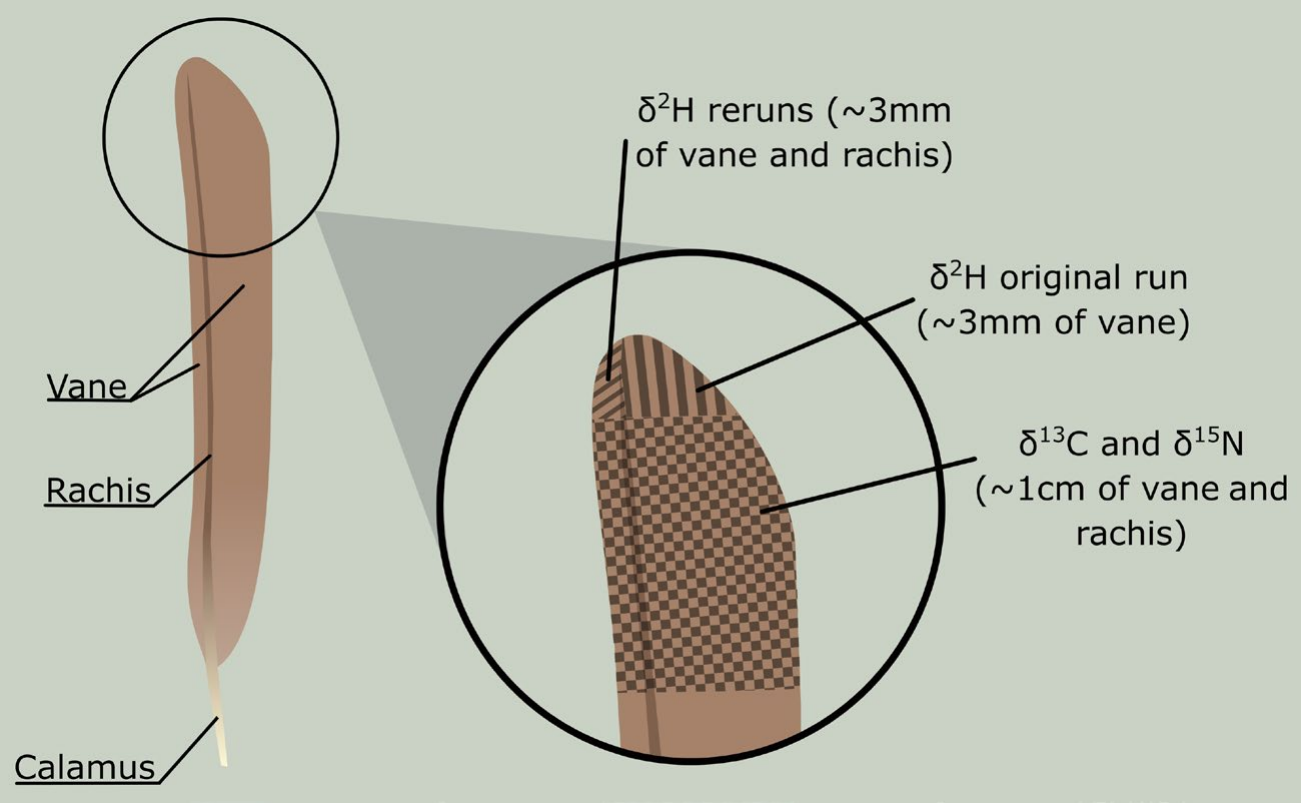
Diagram of feather showing sections that were sampled according to the procedures at Cornell Isotope Laboratory where the feathers were processed. δ^2^H sections represent where the feathers were clipped to process for another stable isotope, hydrogen, which was not a part of this study.

We stored feathers dry in paper envelopes until lab preparation. For stable isotope analysis, we sent rectrices to the Cornell Isotope Laboratory (COIL, Cornell University, Ithaca, NY, USA), where they were cleaned with a 2:1 chloroform:methanol solution to remove surface oils and dried under a fume hood for 24 h. Both samples and standards (see below) were allowed to equilibrate with the COIL lab atmosphere for at least 3 days prior to isotope measurements. Feathers were cut, using the distal section of the vane for δ^13^C and δ^15^N measurements (Figure 2). As feathers are very difficult to homogenize, they are not ground up, and approximately 1 mg of feather material was packed into tin capsules. The isotope measurements were performed using a NC2500 elemental analyzer. COIL analyzed lab standards every 10 samples to measure accuracy and precision, and for both δ^13^C and δ^15^N, the lab standards were ground deer hair (DEER) and Methionine (see Appendix A for delta values and error for the standards). COIL performed corrections using a linear regression and two additional in‐house standards: ground corn leaves (KCRN) and ground Cayuga Brown Trout muscle (CBT), which are calibrated every 6 months against international standards (Vienna Pee Dee Belemnite [VPDB] for δ^13^C and Atmospheric Air for δ^15^N). Feathers were processed in different batches. For δ^13^C, precision was reported as the standard deviation of the internal animal standard (DEER) and given in three separate batches: ±0.03‰ (*n* = 124 feathers), 0.04‰ (*n* = 47 feathers) and 0.08‰ (*n* = 3 feathers). For δ^15^N, precision based on the internal animal standard (DEER) in three separate batches was ±0.04‰ (*n* = 124), 0.03‰ (*n* = 47), and 0.1‰ (*n* = 3; Appendix A). Feather isotope ratios (δ^13^C_f_, δ^15^N_f_) are in delta notation, in units of parts per thousand (‰) normalized to their primary reference scales.

### 2.4 Data Analysis

Values of δ^15^N in the surrounding environment (baseline δ^15^N) are required to estimate the trophic position of a consumer (Post 2002). It was not within the scope of this study to collect baseline information at the known breeding sites of Golden‐crowned Sparrows in this study, which were scattered across remote areas of Alaska and British Columbia (see Figure 1 and Iverson et al. 2024), and for many individuals with feather samples, breeding sites were unknown. Despite this limitation, baseline δ^15^N values were not expected to vary substantially across the Golden‐crowned Sparrow breeding range because nitrogen residence times are assumed to be similar within ecosystem types, and our geographic region of interest has similar precipitation and temperature patterns (see Bowen and West 2019 for a predicted plant nitrogen isoscape). However, to account for differences in breeding locations or timing that could drive differences in δ^15^N_f_ or δ^13^C_f_ (which could indicate differences in foraging habitat type), we conducted a linear regression using the *stats* package in R (R Core Team 2024) including predictors of sex, year, breeding latitude, elevation (*elevatr* package; Hollister et al. 2023), and distance to shoreline (*rnaturalearth* package; Massicotte et al. 2023) using the centroids of known breeding season home ranges for 9 females and 9 males (Iverson et al. 2024). Continuous response and predictor variables for the δ^15^N_f_ analysis were transformed to the log scale prior to analysis to meet assumptions of normality with the small sample size.

Our known breeding locations do not span the entire potential breeding range (Figure 1) and many samples came from birds with unknown breeding locations. Previous work has suggested moderate to strong regional migratory connectivity between breeding and wintering regions for Golden‐crowned Sparrows (Cormier et al. 2016; Pandolfino and Douglas 2021). Therefore, for birds without known breeding locations, we considered the feather collection location (wintering area) as a possible proxy for differing breeding areas, though we cannot know with certainty how well the wintering feather collection locations represent differences in breeding location. Specifically, for all feather samples, to compare δ^15^N_f_ and δ^13^C_f_ for the different sexes, we compared a set of linear regression models (Table 1) using the *stats* and *AICcmodavg* packages in R (R Core Team 2024; Mazerolle 2023), with sex, year, and the feather collection/wintering location as predictors (two outliers removed for the δ^13^C_f_ analysis). We compared all combinations of predictors, including three univariate models and one full model, for a total of seven models for each response. For all linear regressions, no continuous variables were correlated greater than ±0.5, and we checked that all assumptions were met for errors and residuals. Models were averaged when Δ AICc < 2.0, and we report the full model average.

**TABLE 1:** List of models compared for variation in δ^15^N and δ^13^C in feather samples (rectrices) of Golden‐crowned Sparrows ( *Zonotrichia atricapilla* ).

Models	Δ AICc	Weight
**δ** ^**15**^ **N models**		
Sex + year*	0.00	0.39
Sex + collection location*	1.58	0.18
Sex + year + collection location	2.26	0.13
Sex	2.82	0.10
Collection location	3.20	0.08
Year	3.44	0.07
Year + collection location	3.56	0.07
**δ** ^**13**^ **C models**		
Year*	0.00	0.49
Sex + year*	1.41	0.24
Collection location	2.65	0.13
Sex + collection location	4.83	0.04
Collection location + year	5.04	0.04
Sex	5.07	0.04
Sex + year + collection location	7.32	0.01

*Note:* Feathers were collected on wintering grounds from 2019 to 2021 and are expected to reflect diet on breeding grounds. Year = the year of feather collection; Collection location = the wintering grounds of the individual where feathers were collected. Models with asterisks were averaged.

## 3 Results

**FIGURE 3:**
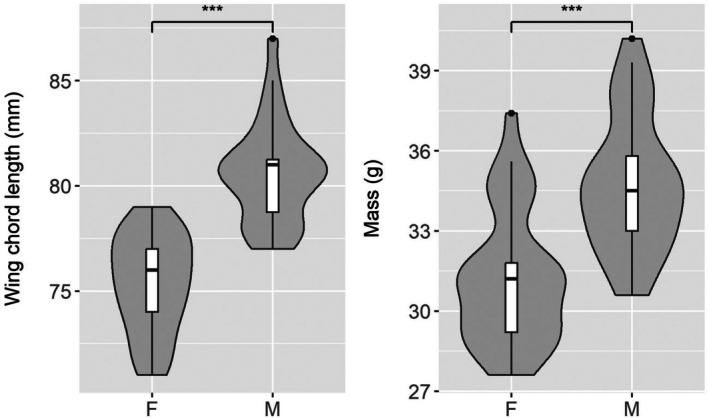
Violin plots of wing chord (mm) and body mass (g) differences by sex (F = female, M = male) for Golden‐crowned Sparrows ( *Zonotrichia atricapilla* ) measured on wintering sites ranging from British Columbia to California. The white boxes represent the interquartile range and the line within it is the median. Areas with wider gray shading represent values with a higher probability to occur in the population. Wing measurements include 31 females and 28 males. Mass measurements include 31 females and 29 males. Both wing chord and mass were significantly different between the sexes (denoted by asterisks). Only individuals whose sex was determined genetically are included.

Genetic sex determination for 60 individuals included 31 females (F) and 29 males (M) including 8:10 F:M from PABC, 5:13 F:M from UCD, 5:0 F:M from HAGM, and 13:6 F:M from ZUMA. Genetically sexed females (*n* = 31) were on average lighter than males (*n* = 29; average mass of females = 31.2 g and males = 34.7 g; *F*(1, 58) = 29.1, *p* < 0.001; Figure 3). We found that males (*n* = 28 because one was missing a wing chord length value) had an average wing chord length of 80.6 mm (range = 77–87 mm) and females (*n* = 31) had an average wing chord length of 75.3 mm (range = 71–79 mm), with males having significantly longer wing chords than females (*F*(1, 57) = 75.19, *p* < 0.001; Figure 3). Using wing chord lengths, we further classified another 13 birds (11 females; 2 males) for a total of 73 sexed individuals including 42 females and 31 males.

For GPS‐tagged birds, the overall δ^15^N_f_ model results were not significant (*F*(5, 12) = 1.767, *p* = 0.19, Adj *R* ^2^ = 0.18) possibly due to our low sample size or due to missing factors in our analysis that would explain variation in δ^15^N_f_. However, the model showed that males had significantly lower δ^15^N_f_ values than females (*p* < 0.05), after accounting for year, latitude, elevation, and distance to shoreline. Similarly, the δ^13^C_f_ model results were not significant (*F*(5, 12) = 2.153, *p* = 0.13, Adj *R* ^2^ = 0.25) but this model had no significant predictors.

**FIGURE 4:**
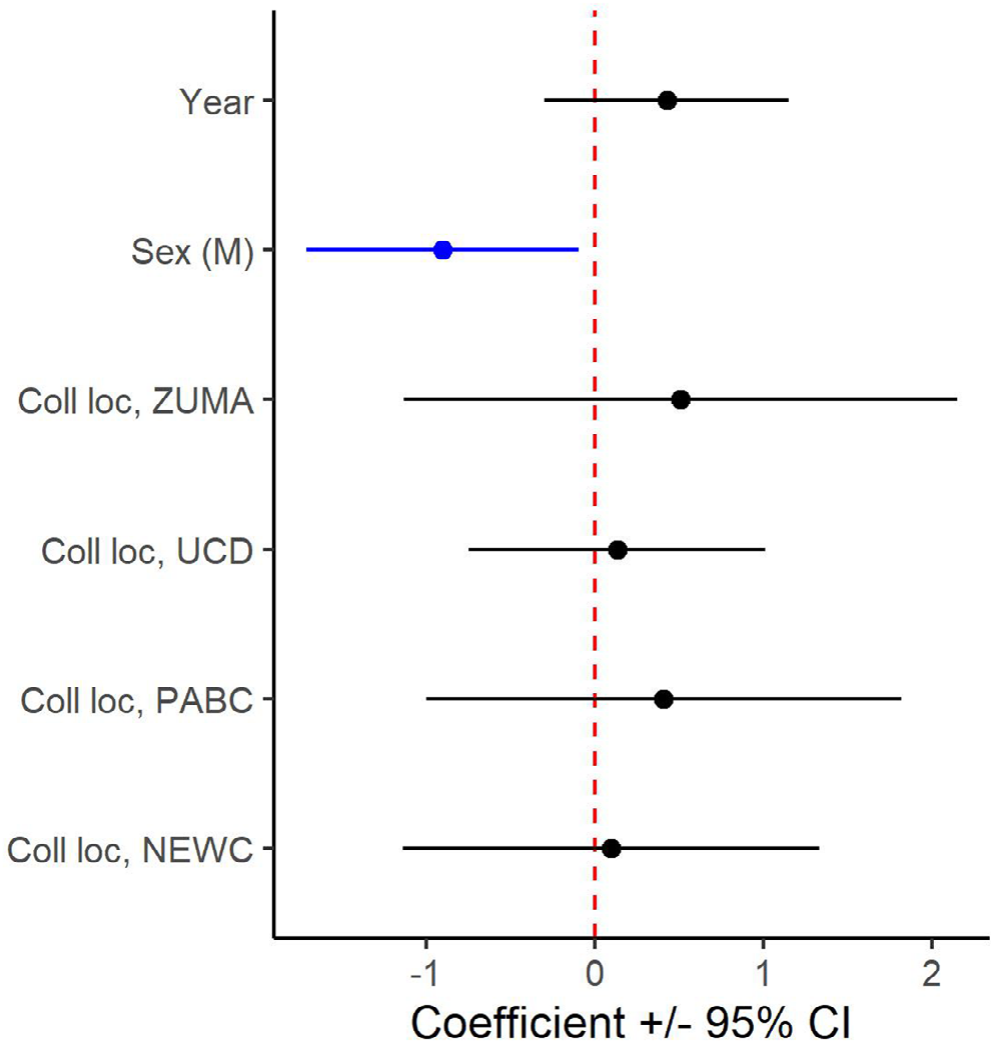
Parameter coefficients (with 95% confidence intervals [CI]) of full averaged model for feather δ^15^N of Golden‐crowned Sparrows ( *Zonotrichia atricapilla* ) sampled on wintering grounds from 2019 to 2021 (*n* = 73). Stable isotope values are expected to be representative of the breeding grounds where the species is most likely to molt. Year represents the year of feather collection, and collection locations are described in detail in Methods and shown in Figure 1.

**FIGURE 5:**
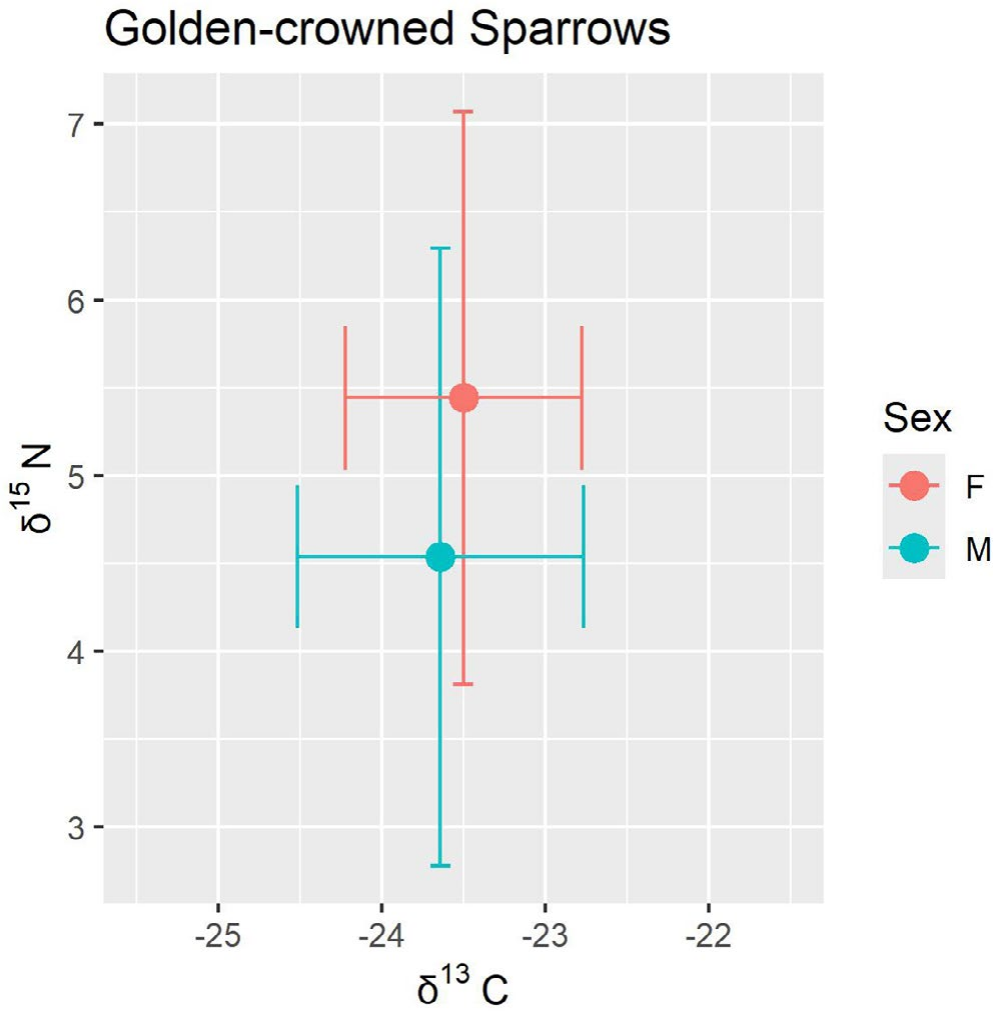
Mean and standard deviation of feather δ^15^N and δ^13^C values representative of the breeding grounds (where the species is most likely to molt) by sex (F = female, M = male) for Golden‐crowned Sparrows ( *Zonotrichia atricapilla* ). Values of δ^15^N_f_ (*n* = 73) include individuals given a sex based on wing chord, and values for δ^13^C_f_ (*n* = 71) include individuals given a sex based on wing chord after removing two genetically sexed birds due to outlier values for δ^13^C_f_. Values of δ^15^N_f_ were significantly different between the sexes, and values of δ^13^C_f_ were not.

Using isotope information from all 73 birds sampled in the northern, central, and southern portions of the wintering range, the significant difference in δ^15^N_f_ by sex remained. Results from the full average model (Table 2) which averaged a model with sex and year (ΔAICc = 0) and a model with sex and collection location (ΔAICc = 1.58) indicated that females had significantly higher δ^15^N_f_ than males (*p* < 0.05) after accounting for year and collection location (both of which were not significant in the full average model; Figure 4). The average δ^15^N_f_ value for females on the breeding grounds (5.4‰ ± 1.63‰, range 2.1‰–9.2‰) was about 0.9‰ higher than the average value for males (4.5‰ ± 1.76‰, range 1.1‰–8.1‰; Figure 5). Average δ^15^N_f_ values for genetically sexed individuals only (*n* = 60) showed a slightly higher difference of 1.2‰, with an average female value of 5.7‰ and an average male value of 4.5‰ (same ranges as above).

**TABLE 2:** Coefficients, *z* values, and *p*‐values of full average models for stable isotope values (δ^15^N and δ^13^C) obtained from feather samples (rectrices) of Golden‐crowned Sparrows ( *Zonotrichia atricapilla* ).

Parameter	Coefficient	*p*
***δ*** ^***15***^ ***N model***		
Intercept	−856.68	0.25
Sex (male)	−0.90	0.03*
Year	0.43	0.25
Collection location (PABC)	0.41	0.57
Collection location (NEWC)	0.10	0.87
Collection location (UCD)	0.13	0.77
Collection location (ZUMA)	0.51	0.54
***δ*** ^***13***^ ***C model***		
Intercept	−665.71	0.01*
Year	0.32	0.02*
Sex	−0.05	0.68

*Note:* Feathers were collected on wintering grounds from 2019 to 2021 and expected to reflect diet on breeding grounds. Year = the year of feather collection; Collection location = the wintering grounds of the individual where feathers were collected. Detailed information about collection locations are given in the Methods and they are also shown Figure 1. Significant *p*‐values (i.e., < 0.05) are denoted by asterisks.

We did not find a difference in δ^13^C_f_ values between males and females (*n* = 71 [two outliers removed]) with mean δ^13^C_f_ values similar for females (−23.5‰ ± 0.7‰) and males (−23.6‰ ± 0.9‰; Figure 5), indicating that there was no evidence for a difference by sex in foraging habitat type. Results from the full average model (Table 2) which averaged a model including only year (ΔAICc = 0) and a model with sex and year (ΔAICc = 1.41) indicated that δ^13^C_f_ significantly increased across the feather collection years of 2019–2021 by an average of 0.3‰ (*p* < 0.05), but sex was not significant (*p* = 0.68).

## 4 Discussion

We found that female Golden‐crowned Sparrows had higher δ^15^N_f_ values on average compared to males. Differences in δ^15^N in populations can arise from different baseline environmental δ^15^N values, physiological processes, or diet differences. Without the baseline environmental values of δ^15^N at each breeding site, we cannot say with certainty how spatially different δ^15^N values would be across the breeding range; however, δ^15^N is not expected to vary substantially across the Golden‐crowned Sparrow breeding range (Bowen and West 2019) and we found that sex was still a significant predictor of δ^15^N_f_ values for birds with known breeding sites after considering year, latitude, elevation, and distance to the shoreline of their breeding locations. Sex was also a significant predictor when considering samples from birds with unknown breeding locations, but because our known breeding sites cover only a portion of the possible breeding range, we cannot rule out that variations in baseline δ^15^N across the larger area could be driving these differences. However, it seems unlikely that males would consistently be found in areas with lower baseline δ^15^N than females because feathers are grown after breeding (when males and females have been paired and in close geographic proximity), and there is no evidence of large post‐breeding movements before migration (Iverson et al. 2024). We also did not find evidence that sex played an important role in differences in foraging habitat type based on the similar δ^13^C_f_ between the sexes—though our finding of significant small increases in δ^13^C_f_ each year may indicate increasingly water‐stressed habitats (Winter 1981). Together, this supports the notion that baseline δ^15^N at their breeding sites is likely not driving the differences found in δ^15^N_f_ between the sexes. To confirm whether our results are due to baseline δ^15^N differences between males and females, further research could gather diet samples across the breeding range or use compound stable isotope analysis (which can differentiate between source and trophic amino acids [Ohkouchi et al. 2017] but is more costly).

We considered the possibility that the shift in δ^15^N_f_ is due to physiological changes for the female during reproduction. Only Golden‐crowned Sparrow females incubate and brood, though both parents feed nestlings, and males have been observed feeding females on the nest (Hendricks 1987). Some large birds use stored protein, primarily from flight muscles, to produce eggs, though for small passerines most of the nutrients required for egg laying must come from foraging during the egg‐formation period (Meijer and Drent 2008), and the use of local dietary sources for egg production has been confirmed using stable isotopes for a small migratory passerine (American Redstart, Langin et al. 2006). Using stored protein could theoretically cause elevated δ^15^N values in tissues, as the protein “consumed” would have a higher δ^15^N value than found in the diet (i.e., they are “eating” themselves). This has been shown in animals under nutritional stress where the δ^15^N values increase as lean body mass decreases (e.g., in Japanese Quail *Coturnix japonica* and Ross' Geese *Anser rossii*, Hobson et al. 1993). It is possible that female Golden‐crowned Sparrows experience nutritional or water stress while incubating and brooding despite being fed by males, however increasing δ^15^N values under nutritional stress may not be applicable to passerines, as an increase in δ^15^N was not seen in feathers or other tissues of malnourished Song Sparrows ( *Melospiza melodia* ) compared to control birds, despite poorer growth and brain development (Kempster et al. 2007).

Another consideration regarding the possibility of stored protein use during reproduction is that for this to account for the increased δ^15^N values in females, it would need to have temporally overlapped with molting, as the values of δ^15^N_f_ we observed were established while rectrices were growing. In the closely related Mountain White‐crowned Sparrow ( *Zonotrichia leucophrys oriantha* ), molting was found during all stages of nesting, but only a small proportion of females (8%) began to molt during incubation (Morton and Morton 1990). Generally, the prebasic molt for Golden‐crowned Sparrows is most likely to occur post‐breeding in line with most songbirds and before the onset of migratory restlessness (King and Farner 1963), with rectrices replaced relatively late in the molt (once primaries have reached p5–p7; Pandolfino et al. 2025). Therefore, while it is not definitive that they would have a full separation of breeding and molt (Howell 2010; Willoughby 1991), new feathers (and especially rectrices) for adult females would likely be grown from locally acquired food instead of stored protein, since the overlap is far less likely to occur during the early stages of breeding. Given these considerations, we suggest that the higher δ^15^N_f_ we observed in females is unlikely to be a result of the physiological effects of nesting though further research could be useful on whether the female experiences nutritional stress or other physiological effects of reproduction that could be related to increased δ^15^N_f_.

The differences we found in δ^15^N_f_ values (on average ~1‰ between the sexes) are therefore most likely caused by sex‐based differences in foraging during molting on the breeding grounds. Though there is evidence that some passerine species experience habitat segregation on the wintering grounds (Cooper et al. 2021; Marra and Holmes 2001), this sex‐based difference in foraging during the breeding period has not been found for similar sparrow species; that is, isotopic studies of wild Song Sparrows and Fox Sparrows showed no differences between males and females in blood δ^15^N values during breeding (Hipfner et al. 2013).

Little is known about Golden‐crowned Sparrow adult diets on the breeding grounds, and at other times of the year, they are omnivores, consuming primarily (97%–99%) plant matter. However, the small percent of insects recorded in stomachs during the nonbreeding season included Hymenoptera (ants, bees, and wasps), Lepidoptera (moths and butterflies), Coleoptera (beetles), and Diptera (crane flies; Beal 1910, Martin et al. 1951). During the breeding season, there is no information on adult diet but both parents feed nestlings arthropods, including lepidopteran adults and larvae, crane flies, and stoneflies (Hendricks 1987; Pandolfino et al. 2025). Differences in foraging needs between the adults on the breeding grounds could arise if females attempt to replace lost nutrients from nesting prior to initiating migration, potentially consuming more arthropods (or switching to more predatory arthropods) than males during this time, which would result in a higher δ^15^N_f_ signal than plant material (or herbivorous arthropods). Because we do not have δ^15^N values for their food items, we cannot say with certainty what food items the birds were targeting. As a comparison, studies of δ^15^N in other sparrow species (Lincoln's Sparrow and White‐crowned Sparrow) and their potential prey showed arthropods had ~0‰–4‰ less δ^15^N than the bird δ^15^N signatures (and sometimes higher in the case of spiders) and plant material was ~5‰–7‰ less than the bird δ^15^N signatures (adult bird δ^15^N values were between ~6‰ and 8‰; Beaulieu and Sockman 2012). Looking at similar species (which may be more likely to have similar discrimination factors) as a comparison, the study looking at the differences in diet between Fox Sparrows and Song Sparrows found a difference of ~1.5‰, which they concluded was likely due to Song Sparrows consuming more animal than plant food in their diet than Fox Sparrows (Hipfner et al. 2013) and the significant difference in diets between Lincoln's Sparrows and White‐crowned Sparrows was due to ~1‰–1.5‰ difference in δ^15^N. The differences of ~1‰ in δ^15^N_f_ that we found between the sexes for Golden‐crowned Sparrows similarly suggest that females may be consuming more animal than plant food in their diet than males.

Further investigation in the field could yield more insight through observation and by gathering δ^15^N values for potential food items to improve our understanding of sex‐based diet differences for Golden‐crowned Sparrows. Also, as Golden‐crowned Sparrows are sexually monomorphic, adding additional measurements such as tail length can help assign sex when combined with wing chord measurements (Pyle 2022) if genetic tests or information from breeding grounds (i.e., brood patch or cloacal protuberance) are not available or feasible to obtain. The sex‐based differences we found in this sexually monomorphic migratory passerine add to the list of avian species with differential foraging patterns by sex. Also, if females are reliant on insects during the breeding season, then the success of this species will be tied to insect populations, which are currently experiencing large declines across the world (Wagner 2019).

## Author Contributions

**Autumn R. Iverson:** conceptualization (lead), data curation (lead), formal analysis (lead), funding acquisition (lead), investigation (lead), methodology (lead), project administration (equal), resources (equal), supervision (equal), writing – original draft (lead), writing – review and editing (lead). **Renée L. Cormier:** conceptualization (supporting), data curation (supporting), investigation (supporting), methodology (supporting), project administration (equal), resources (equal), supervision (supporting), writing – review and editing (supporting). **Diana L. Humple:** conceptualization (supporting), data curation (supporting), funding acquisition (supporting), investigation (supporting), methodology (supporting), project administration (equal), resources (equal), supervision (supporting), writing – review and editing (supporting). **Thomas P. Hahn:** conceptualization (supporting), investigation (supporting), methodology (supporting), project administration (equal), resources (equal), supervision (supporting), writing – review and editing (supporting). **Jessica Schaefer:** data curation (supporting), investigation (supporting), writing – review and editing (supporting). **Elisha M. Hull:** conceptualization (supporting), project administration (supporting), writing – review and editing (supporting). **Walter H. Sakai:** investigation (supporting), project administration (equal), resources (equal), writing – review and editing (supporting). **Samuelle Simard‐Provençal:** data curation (supporting), investigation (supporting), project administration (supporting), resources (supporting), visualization (supporting), writing – review and editing (supporting).

## Conflicts of Interest

The authors declare no conflicts of interest.

## Data Availability

The data on which the analyses were conducted are available on Dryad at https://doi.org/10.5061/dryad.0000000fz.

## Appendix A

Table showing δ^15^N and δ^13^C delta values and standard deviation (SD) error for lab standards used with batches of feather samples. The number of feather samples (*n* = 174) is greater than the number of individuals sampled (*n* = 162) due to some birds having repeat samples that were not used in the analysis (because they were repeatedly tagged or because they had a sample associated with both a non‐tagged and a tagged period), and three feathers being processed twice in the lab to double‐check values (Batch 3). There were two runs for Batch 1, indicated by 1.1 and 1.2. Information about how standards were used can be found in the Methods. Acronyms for standards are as follows: CBT, Cayuga Brown Trout muscle; DEER, ground deer hair; KCRN, ground corn leaves.

**Table:**

Batch	Samples	Standard	δ15N vs. At. Air (mean ± SD)	δ13C vs. VPDB (mean ± SD)
1.1	49	CBT	17.49 ± 0.03	−25.58 ± 0.04
		KCRN	1.27 ± 0.06	−13.01 ± 0.04
		DEER	6.27 ± 0.04	−19.97 ± 0.02
1.2	75	CBT	17.49 ± 0.05	−25.58 ± 0.03
		KCRN	1.27 ± 0.07	−13.01 ± 0.04
		DEER	6.24 ± 0.04	−19.99 ± 0.04
1		Methionine	−0.08 ± 0.23	−27.08 ± 0.08
2	47	CBT	17.49 ± 0.07	−25.58 ± 0.04
		KCRN	1.27 ± 0.12	−13.01 ± 0.02
		DEER	6.29 ± 0.03	−19.97 ± 0.04
		Methionine	−0.15 ± 0.26	−26.83 ± 0.30
3	3	CBT	17.49 ± 0.16	−25.58 ± 0.14
		KCRN	1.27 ± 0.22	−13.02 ± 0.08
		DEER	6.56 ± 0.10	−20.12 ± 0.08
		Methionine	−0.48 ± 1.00	−26.97 ± 0.25
